# Supplementary material for: Mechanisms of Cannabis Growth Promotion by Bacillus velezensis S141
Source: Plants (Basel). 2024 Oct 24;13(21):2971. doi: 10.3390/plants13212971 (PMC11547760; doi:10.3390/plants13212971)
Supplement: Supplementary file 1 [file plants-13-02971-s001.zip › plants-3123197-supplementary.pdf]

Supplementary data

Unraveling the Mechanisms of Cannabis Growth Promotion by *Bacillus velezensis* S141

Phirom Aunkam[1], Surachat Sibponkrung[2], Sirawich Limkul[1], Tuangrak Seabkongseng [1], Kanjana Mahanil[3], Kamolchanok Umnajkitikorn[4], Nantakorn Boonkerd[1], Neung Teaumroong[1], Shusei Sato[5], Panlada Tittabutr[1], and Pakpoom Boonchuen[1,*]

[1] School of Biotechnology, Institute of Agricultural Technology, Suranaree University of Technology, Nakhon Ratchasima 30000, Thailand.
[2] Center of Excellent in Agricultural Product Innovation, Suranaree University of Technology, Nakhon Ratchasima 30000, Thailand.
[3] Institute of Research and Development, Suranaree University of Technology, Nakhon Ratchasima 30000, Thailand.
[4] School of Crop Production Technology, Institute of Agricultural Technology, Suranaree University of Technology, Nakhon Ratchasima, 30000, Thailand
[5] Graduate School of Life Sciences, Tohoku University, Sendai, 980-8577, Japan.
***** Correspondence: author E-mail: pakpoom.b@sut.ac.th

Table S1 Bacterial strains used in this study.

**Bacterial Strains**	**Relevant Genotype or Description**	**References**
Bacillus velezensis S141	*Wild type*	Sibponkrung, S.; Kondo, T.; Tanaka, K.; Tittabutr, P.; Boonkerd, N.; Yoshida, K.-i.; Teaumroong, N. Co-inoculation of *Bacillus velezensis* strain S141 and *Bradyrhizobium* strains promotes nodule growth and nitrogen fixation. Microorganisms 2020, 8, 678.
B. velezensis S141Δ*dhaS*	*dhaS deletion, ΔdhaS::erm*^{r}	
B. velezensis S141Δ*yhcX*	*yhcX deletion, ΔyhcX::kan*^{r}	
B. velezensis S141Δ*IPyAD*	*IPyAD deletion, ΔIPyAD::spm*^{r}	
B. velezensis S141Δ*ipt*	*IPT deletion, Δipt::phle*^{r}	
B. velezensis S141Δ*ipi*	*IPI deletion, Δipi::kan*^{r}	

Table S2 The greenhouse experimental conditions.

**Conditions**	**Non-treated soil normal fertilizer**	**Non-treated soil lower fertilizer**	**Boiled water-treated soil normal fertilizer**	**Boiled water-treated soil lower fertilizer**
Pretreatment	-	-	Portions of the soil were subjected to addition by pouring through the soil volumes of boiled water (100 ºC) at a ratio of 0.5 L boiled water per 1 kg of soil<br>**References**<br>Saied, M., *Evaluation of hot water soil treatment against cucumber root rot disease under greenhouse conditions.* Research Journal of Agriculture and Biological Sciences, 2011. **7**(2): p. 212-222.	
Planting materials	- Loam soil<br>-Coco husk chips<br>-Rice husk-based charcoal<br>-Manure			
plant pots	-Pot Size 30 L<br>-Pot Diameter (Base) 13 inches<br>- Pot Diameter 17 INCHES<br>-Pot Height 15 INCHES			
Watering	every morning 1.5 - 2 L / plant			
Humidity	- 40 - 60%<br>- 9 - 11 hr.			
Temperature	30 - 37 °C			
Urea Fertilizer 46-0-0-0	10 g / plant / week			
AB Fertilizer EC:1.8-2 mS/cm pH 6.5 - 7	2 L / 2 times / week	1 L / 2 times / week	2 L / 2 times / week	1 L / 2 times / week

Table S3 Elemental analysis of planting material

Sample	EC (ds/m) 1:5	pH 1:5	% OM	% N	% P	% K	% Ca	% Mg
Planting material	2.21	6.84	33.66	1.18	0.72	0.08	1.56	0.62

Table S4 Summary of primers for qRT-PCR used in this study.

Primers	Accession	Sequence (5′to3′)	Annealing temperature (◦C)	Experiment
Actin -F	XM_030632129.2	TTGCTGGTCGTGATCTTACTG	60	Internal control
Actin -R		GTCTCCATCTCCTGCTCAAAG	60	
THCAS-F	XM_030649882.2	GCTCTCTTCGTTGCTGGACT	60	qRT-PCR
THCAS-R		TGTTCCCACCTCTATGCCCA	60	
CBDAS-F	XM_030623918.2	CTTAGTTTGGCGGCTGGGTA	60	
CBDAS-R		CTTTGGGACAGCAACCAGTCT	60	
SAUR50-F	XM_030643469.2	GCTCTCTTCGTTGCTGGACT	60	
SAUR50-R		TGTTCCCACCTCTATGCCCA	60	
XEHP25-F	XM_030654082.2	CTTAGTTTGGCGGCTGGGTA	60	
XEHP25-R		CTTTGGGACAGCAACCAGTCT	60	
GLP2-1-F	XM_030625170.2	AGGCCTTGGGACTTGCTTTC	60	
GLP2-1-R		GACCGTGTACAAGAGCAGCT	60	
ABC29-F	XM_030635527.2	TGGATGGTGCTCCTTTTCTTCG	60	
ABC29-R		AGGCTCCAGACCAAATCCCA	60	
CBL120-F	XM_030646708.2	TGTGTGGACTCCTCAACTCCA	60	
CBL120-R		ACTCAAACATGCGACCACGT	60	
GTLS-F	XM_030629393.2	AGCTGTGACGGGTCAACTTC	60	
GTLS-R		AGAGATTGGGCCGATTGGTG	60	
LRLK4-F	XM_061114175.1	AGAAGCTAAGGCACCTGCAG	60	
LRLK4-R		CGGTATGGACTTGGTGCAGT	60	
PRP-1A-F	XM_030629932.1	TGGATGGTGCTCCTTTTCTTCG	60	
PRP-1A-R		AGGCTCCAGACCAAATCCCA	60	
EIX1-F	XM_030640350.1	TGTGTGGACTCCTCAACTCCA	60	
EIX1-R		ACTCAAACATGCGACCACGT	60	
CRF5-F	XM_030632056.1	AGCTGTGACGGGTCAACTTC	60	
CRF5-R		AGAGATTGGGCCGATTGGTG	60	
CAO -F	XM_030634090.1	AGAAGCTAAGGCACCTGCAG	60	
CAO -R		CGGTATGGACTTGGTGCAGT	60	
IAA-2-F	XM_030653759.2	GACACTTGGTGGTTTGCG	60	
IAA-2-R		TGCCCGAGTTACCTGAAT	60	
ARR5-F	XM_030639807.2	CAGGGATGACAGGATATGAGCTTC	60	
ARR5-R		GACGATTGTGACGACAACACG	60	
ARR12-F	XM_030637161.2	TGTGTGGACTCCTCAACTCCA	60	
ARR12-R		ACTCAAACATGCGACCACGT	60	
UDP -F	XM_030628925.1	TGGATGGTGCTCCTTTTCTTCG	60	
UDP -R		AGGCTCCAGACCAAATCCCA	60	
ERFC3 -F	XM_030647537.1	TGTGTGGACTCCTCAACTCCA	60	
ERFC3 -R		ACTCAAACATGCGACCACGT	60	
S141-F	AP018402	TGATTGCCGGCACAGAAAATAACAGG	60	Bacterial copy number
S141-R		GGTTTCCGGTACCACGTCTGTC	60	

Table S5. Sequencing data analysis RNA sequencing (RNA-Seq) was analyzed and indicated of percent of GC, Q20 and Q30 of each sample.

#SampleID	Total Reads	Mapped Reads	GC (%)	Q20(%)	Q30(%)
CBRC1	41,813,660	37,584,995 (89.89%)	43.40	97.89	95.97
CBRC2	40,907,318	37,014,927 (90.48%)	43.63	97.82	95.78
CBRC3	40,852,224	36,650,102 (89.71%)	43.71	96.72	94.24
CBRI1	46,624,092	41,736,266 (89.52%)	43.59	96.88	94.54
CBRI2	42,244,200	37,838,633 (89.57%)	43.56	96.87	94.45
CBRI3	41,335,878	37,093,945 (89.74%)	43.53	96.80	94.39
